# Health-related quality of life (FACT-GP) in Sweden

**DOI:** 10.1186/s12955-020-01420-1

**Published:** 2020-06-08

**Authors:** Ann-Sophie Lindqvist Bagge, Anders Carlander, Claudia Fahlke, Roger Olofsson Bagge

**Affiliations:** 1 Department of Psychology, University of Gothenburg, Box 500 Gothenburg, Sweden; 2 Wallenberg Centre for Molecular and Translational Medicine, University of Gothenburg, Gothenburg, Sweden; 3 SOM Institute, University of Gothenburg, Gothenburg, Sweden; 4 Department of Surgery, Sahlgrenska University Hospital, Region Västra Götaland, Gothenburg, Sweden; 5 Department of Surgery, Institute of Clinical Sciences, Sahlgrenska Academy at the University of Gothenburg, Gothenburg, Sweden

**Keywords:** Health-related quality of life, HRQOL, The functional assessment of cancer therapy - general population, FACT-GP, Self-rated health, SRH, Normative data

## Abstract

**Background:**

Many studies have used disease-specific instruments, such as the Functional Assessment of Cancer Therapy (FACT), when studying health-related quality of life (HRQOL) in patients. Few studies however, have described normative HRQOL values in the general population using FACT - General Population (FACT-GP). The general aim of the present study is thus to describe the normative HRQOL values in the general Swedish population by using the FACT-GP instrument and to investigate to what degree sociodemographic factors and status of self-rated health (SRH) correlate with HRQOL.

**Methods:**

The participants consisted of a pre-stratified (gender, age and education) sample of Swedish citizens that previously had enrolled to be a part of a web panel hosted by a research institute (SOM Institute) at the University of Gothenburg in Sweden. The HRQOL was assessed by using the FACT-GP and SRH.

**Results:**

A higher FACT-GP score was mainly associated with males, higher age, higher income and better SRH. The results showed that the Swedish sample scored lower on FACT-GP than previous studies.

**Conclusions:**

Since HRQOL is frequently used as an important endpoint in healthcare research, there is an increasing need for normative data. The results from this study serve as a general population standard against which other studied HRQOL-data could be evaluated.

## Introduction

During the last decades there has been an increasing trend in clinical studies to use patient reported outcomes (PROs) in addition to clinical outcomes. PRO is defined as any “report of the status of a patient’s health condition that comes directly from the patient, without interpretation of the patient’s response by a clinician or anyone else” [1]. Health-related quality of life (HRQOL) is a commonly used PRO, where HRQOL is commonly seen as a multidomain concept that represents the patient’s general perception of the effect of illness and treatment on physical, psychological and social aspects of life [1–3]. HRQOL is associated with sociodemographic factors such as gender [4], age [4, 5], level of income and education [6]. It has also been shown that there is a correlation between self-reported HRQOL and self-rated health (SRH) status [7, 8].

The instrument *Functional Assessment of Cancer Therapy – General* (FACT-G) was developed in 1987 and the first validation study was published in 1993 [9]. FACT-G is a 27-item self-administered instrument divided into four basic domains of self-perceived HRQOL; Physical well-being (PWB), Social/family well-being (SWB), Emotional well-being (EWB) and Functional well-being (FWB) [9]. To the generic core set (FACT-G), different disease-specific subscales are added, mainly focusing on different types of cancer. FACT-G, together with the disease-specific subscales, are referred as the FACT-instruments.

Today there are over 90 different disease-specific FACT-instruments and these have been translated and linguistically validated in 70 different languages. The FACT-G has undergone psychometric testing and, in general, has good psychometric properties. Reliability and validity, including responsiveness, have been well documented in several clinical settings and in clinical trials [8–10]. The test-retest reliability coefficients range from 0.82 to 0.92 and internal consistency of subscales ranges from 0.60 to 0.83 [9, 11]. By removing six disease-related questions from the 27-item FACT-G, the instrument can be converted to address the general population, i.e. *Functional Assessment of Cancer Therapy – General Population* (FACT-GP).

Though many studies have used disease-specific FACT-instruments when studying HRQOL in patients, few studies have described normative HRQOL scores in a general population using either FACT-G or FACT-GP [4, 7, 12]. Even if HRQOL data for cancer and chronically ill patients are useful when evaluating or comparing treatment regimes, it is also important that the results can be interpreted with respect to a general population. However, few studies have described normative HRQOL scores in the general population using the commonly used FACT-instruments. Since HRQOL scores have been associated with sociodemographic factors [5–7, 13], the main aim of the present study was to describe the HRQOL scores in a general Swedish population by using the FACT-GP instrument and to investigate potential relationships with sociodemographic factors as well as with SRH.

## Methods

### Participants and procedure

HRQOL data were collected through a web panel (The Citizen Panel) hosted by a research institute (SOM Institute) at the University of Gothenburg in Sweden. The participants consisted of a pre-stratified (gender, age and education) sample of Swedish adult citizens (> 18 years of age) that previously had enrolled to be a part of the web panel.

### HRQOL

FACT-GP [14] is based on FACT-G [9]. The 27-items of FACT-G convert to FACT-GP by removing 6 disease-related questions. The 21-item FACT-GP subscales and overall summary scores are “pro-rated” (i.e. recoded with the FACT scoring macro: [Sum of item scores] x [N of items in subscale] / [N of items answered]) in order to be comparable to the 27-item FACT-G (see www.facit.org for details and resources on scoring). FACT-GP is based on four basic domains of self-perceived HRQOL; PWB (score range 0–24), SWB (score range 0–20), EWB (score range 0–16) and FWB (score range 0–24), and a total summary score of FACT-GP (score range 0–84) with each item rated on a 5-point Likert scale (ranging from 0 = *not at all* to 4 = *very much*). The pro-rated mean scores of FACT-GP that we use in this research results in a comparable equivalent to the FACT-G score: PWB (score range 0–28), SWB (score range 0–28), EWB (score range 0–24), FWB (score range 0–28) and FACT-GP (score range 0–108). Higher FACT-GP scores indicate a higher level of perceived HRQOL. The T-score (Table 3) conversion is a procedure that will place all the raw scores on a standardized scale with a mean of 50 and standard deviation of 10 thus allowing comparisons between samples and patients. T-scores are calculated as

$$ T\  score=\left[\left(\frac{\left( Raw\  score- Raw\  score\ M\right)}{Raw\  score\  SD}\right)\times 10\right]+50 $$

### SRH

SRH status was assessed by using the single-item question “How do you rate your general health condition?”. The endpoints of the scale are at the bottom (0) “Worst condition you can imagine”, and at the top [10] “Best condition you can imagine”. Apart from being asked to rate their SRH, the participants were also asked if they had ever been diagnosed with cancer [yes/no]. If yes, the participants were asked for how long ago they had received their cancer diagnosis [0–5 yrs./6–10 yrs./11+ yrs].

### Ethical consideration

The study was approved by the regional Swedish Ethical Review Authority (Dnr 948–18) and conducted according to the 1964 Declaration of Helsinki. All participants signed informed consent before entering the study.

## Results

### Participants

An invitation to participate in the study was sent out to 4000 participants based on a non-probability (i.e. self-recruited) sample within the panel. The response rate was 69.6% with 2791 responses collected during 12 days with a minimal number of breakoffs (*n* = 91), partial responses (*n* = 6), or e-mail bounce backs (*n* = 25). One e-mail reminder was sent after 7 days and the entire data collection was carried out in 12 days.

There were an approximately equal number of males (52%) and females (48%). The participants were somewhat older (mean age, M = 48.84, SD = 15.38), had a higher educational level (bachelor degree or higher = 28.7%) and higher income (median range = 30′-37′ SEK) compared to the general population [15]. We applied post-stratification weights based on gender, age and education in order to reflect the distribution of Swedish citizens. However, the results differed only marginally. We decided to proceed with unweighted data since that may be considered more transparent and is associated with less risk of introducing additional skew. For a detailed description of the sample characteristics see Table 1.

**Table 1:** Study population characteristics (*n* = 2791)

	Sample		Sweden^a^
	n	Percent	Percent
Sex			
Female	1266	48%	49.7%
Male	1361	51.7%	50.3%
Age			
18–24	142	5.7%	13.8%^b^
25–34	451	16.2%	17%
35–44	388	13.9%	15.2%
45–54	512	18.3%	16%
55–64	489	17.5%	13.8%
65+	498	17.8%	24.2%
Income			
< 4000 SEK	77	3%	
4–12,999 SEK	268	10.3%	
13–18,999 SEK	267	10.3%	
19–25,999 SEK	356	13.8%	
26–36,999 SEK	752	29%	
37–54,999 SEK	617	23.7%	
> 55,000 SEK	192	7.4%	
Education			
Primary and secondary education	97	3.7%	17.14%
Upper secondary	773	29.4%	42.9%
Post-secondary less than 3 years	1005	38.2%	14.5%
Post-secondary more than 3 years	698	26.6%	21.7%
Post graduate/PhD	56	2.1%	1.1%
Birthplace			
Sweden	2510	95.3%	
other country in Europe	90	3.4%	
outside of Europe	35	1.3%	
Living area			
Large city^c^	999	37.9%	
Large town	739	28.1%	
Town	548	20.8%	
Village/countryside	314	11.9%	
Abroad	34	1.3%	

^a^Statistics Sweden (2018): www.statistikdatabasen.scb.se/pxweb/en/ssd/

^b^Age 15-24

^c^Stockholm, Gothenburg and Malmoe

### HRQOL

FACT-GP pro-rated means (i.e. recoded with the FACT scoring macro), standard deviation, percentage at floor/ceiling (i.e. percentage scoring min/max score), quartiles (25 50 75), skewness, kurtosis and internal consistency (alpha) are displayed in Table 2. Comparing the percentage at the floor and ceiling respectively reveals that there are relatively more participants scoring higher on each subscale. This is further substantiated by the skewness statistic (Table 2) showing that there is a general tendency towards a negatively skewed data distribution which means relatively more higher scores than lower scores.

**Table 2:** Normative data of a Swedish general population sample based on pro-rated mean scores

	PWB (0–28)	SWB (0–28)	EWB (0–24)	FWB (0–28)	FACT-GP (0–108)
Total (N = 2791)					
Mean	22.9	17.5	18.8	18.5	77.7
Standard deviation	4.9	6.6	5.0	6.4	18.5
Percentage at floor	0.1	0.9	0.3	0.2	0.1
Percentage at ceiling	13.7	3.9	21.3	5.3	0.2
Min score	1.17	0.0	0.0	0.0	15.1
25th percentile	21	14.0	16.5	14.0	66.0
50th percentile (median)	24.5	18.2	19.5	19.8	81.0
75th percentile	26.8	22.4	22.5	23.3	92.1
Max score	28	28	24	28	108
Cronbach’s alpha	.82	.81	.77	.88	.92
Males (*n* = 1361)					
Mean	23.8	16.8	19.7	18.7	78.9
Standard deviation	4.4	6.7	4.7	6.3	18.0
Percentage at floor	0.2	1.3	0.3	0.3	0.1
Percentage at ceiling	16.6	2.8	26.5	5.9	0.8
Min score	2.3	0.0	0.0	0.0	15.1
25th percentile	22.2	12.6	18.0	15.2	68.9
50th percentile (median)	24.5	18.2	21.0	19.8	82.0
75th percentile	26.8	22.4	24.0	23.3	92.4
Max score	28.0	28.0	24.0	28.0	108.0
Females (*n* = 1266)					
Mean	22.0^***^	18.3^***^	18.0^***^	18.3	76.6^**^
Standard deviation	5.3	6.4	5.2	6.4	18.9
Percentage at floor	0.1	0.6	0.4	0.2	0.1
Percentage at ceiling	10.5	5.4	16.2	4.7	0.2
Min score	1.2	0.0	0.0	0.0	17.1
25th percentile	18.7	14.0	15.0	14.0	64.2
50th percentile (median)	23.3	19.6	19.5	19.8	79.5
75th percentile	25.7	22.8	22.5	23.3	91.7
Max score	28.0	28.0	24.0	28.0	108.0

*p*-values of pairwise mean comparison between genders: * *p* < .05, ** *p* < .01, *** *p* < .001

T-scores for the Swedish general population sample based on raw score are displayed in Table 3 for standardized comparisons between samples, individuals and patients.

**Table 3:** T-score table for the Swedish general population sample based on raw score

PWB		SWB		EWB		FWB		FACT-GP			
Raw score	T-score	Raw score	T-score	Raw score	T-score	Raw score	T-score	Raw score	T-score	Raw score	T-score
n.a		0	23.7	0	12.4	0	21.2	11	16.8	48	42.0
1	6.1	1	25.9	1	15.4	1	23.0	12	17.4	49	42.7
2	8.5	2	28.1	2	18.4	2	24.9	13	18.1	50	43.4
3	10.9	3	30.2	3	21.4	3	26.7	14	18.8	51	44.1
4	13.2	4	32.4	4	24.4	4	28.5	15	19.5	52	44.7
5	15.6	5	34.6	5	27.4	5	30.3	16	20.2	53	45.4
6	17.9	6	36.7	6	30.4	6	32.2	17	20.8	54	46.1
7	20.3	7	38.9	7	33.4	7	34.0	18	21.5	55	46.8
8	22.7	8	41.1	8	36.4	8	35.8	19	22.2	56	47.5
9	25.0	9	43.2	9	39.4	9	37.6	20	22.9	57	48.2
10	27.4	10	45.4	10	42.4	10	39.5	21	23.6	58	48.8
11	29.8	11	47.6	11	45.4	11	41.3	22	24.3	59	49.5
12	32.1	12	49.7	12	48.4	12	43.1	23	24.9	60	50.2
13	34.5	13	51.9	13	51.4	13	44.9	24	25.6	61	50.9
14	36.9	14	54.0	14	54.4	14	46.8	25	26.3	62	51.6
15	39.2	15	56.2	15	57.4	15	48.6	26	27.0	63	52.2
16	41.6	16	58.4	16	60.4	16	50.4	27	27.7	64	52.9
17	43.9	17	60.5			17	52.2	28	28.4	65	53.6
18	46.3	18	62.7			18	54.1	29	29.0	66	54.3
19	48.7	19	64.9			19	55.9	30	29.7	67	55.0
20	51.0	20	67.0			20	57.7	31	30.4	68	55.7
21	53.4					21	59.5	32	31.1	69	56.3
22	55.8					22	61.4	33	31.8	70	57.0
23	58.1					23	63.2	34	32.5	71	57.7
24	60.5					24	65.0	35	33.1	72	58.4
								36	33.8	73	59.1
								37	34.5	74	59.8
								38	35.2	75	60.4
								39	35.9	76	61.1
								40	36.5	77	61.8
								41	37.2	78	62.5
								42	37.9	79	63.2
								43	38.6	80	63.8
								44	39.3	81	64.5
								45	40.0	82	65.2
								46	40.6	83	65.9
								47	41.3	84	66.6

(See Supplementary Table A1 for normative data of a Swedish general population sample based on pro-rated mean scores of the 21 items).

### Sociodemographic factors

Table 4 displays how each subscale and overall summary FACT mean score differs depending on the reported sociodemographic background variables. There was a statistically significant difference between men (M = 78.9, SD = 18.0) and women (M = 76.6, SD = 18.9) on the overall summary FACT-GP scale [F (1, 2597) = 10.45, *p* = 0.001] and for each subscale (*p* < .001) except for FWB (*p* = 0.10), although the effect size was small (Cohen’s d = 0.12).

**Table 4:** FACT-GP pro-rated mean scores across sample characteristics

	PWB		SWB		EWB		FWB		FACT-GP	
	M	SD	M	SD	M	SD	M	SD	M	SD
Sex										
Female	22.0	5.2	18.3	6.4	18.0	5.2	18.3	5.2	76.6	18.9
Male	23.8	4.4	16.8	6.7	19.7	4.7	18.7	6.3	78.9	18.0
Age										
18–24	23.2	3.9	18.1	6.6	17.4	4.9	18.5	5.7	77.1	16.6
25–34	21.9	5.0	16.4	6.8	17.0	5.3	16.9	6.4	72.1	18.3
35–44	22.3	5.0	16.3	6.6	18.4	4.8	17.3	5.9	74.4	17.5
45–54	22.5	5.5	17.3	6.7	19.0	5.0	18.3	6.5	77.1	19.4
55–64	23.0	5.0	18.2	6.5	19.6	5.0	19.4	6.7	80.1	19.3
65+	24.3	3.9	18.7	6.1	20.2	4.3	20.0	5.8	83.3	16.4
Income										
< 4000 SEK	20.9	5.7	15.5	7.5	16.0	5.1	14.6	7.4	67.1	20.4
4–12,999 SEK	20.8	5.5	16.1	7.2	16.0	5.5	15.7	6.8	68.7	20.0
13–18,999 SEK	21.2	5.6	16.4	7.0	17.3	5.7	15.8	6.7	70.6	20.2
19–25,999 SEK	22.8	4.7	17.1	6.4	18.9	4.8	18.3	6.0	77.0	17.2
26–36,999 SEK	22.8	4.8	17.6	6.5	19.3	4.7	18.7	6.1	78.4	18.8
37–54,999 SEK	24.2	3.8	18.2	6.2	19.8	4.4	20.1	5.5	82.4	16.0
> 55,000 SEK	25.1	3.6	19.3	5.5	20.9	3.7	22.1	4.9	87.3	14.1
Education										
Elementary	21.4	5.3	16.1	6.9	17.4	5.8	16.3	7.2	71.1	20.6
High school	22.8	5.1	17.1	6.9	18.8	5.2	18.2	6.6	76.9	19.4
Vocational/associate/other	23.1	4.6	17.4	6.6	19.4	4.7	18.8	6.0	78.8	17.6
College < 3 years	22.8	5.0	17.3	6.7	18.9	5.1	18.2	6.5	77.3	19.2
College > 3 years	23.0	4.8	18.2	6.1	18.6	4.8	19.0	6.0	78.8	17.3
Graduate/PhD	24.2	3.6	18.0	5.2	19.2	4.5	19.9	5.3	81.2	12.8
Birthplace										
Sweden	22.9	4.9	17.5	6.5	18.9	5.0	18.5	6.3	77.7	18.4
other country in Europe	22.6	5.2	16.7	7.3	18.6	5.4	18.1	6.9	75.9	21.2
outside of Europe	23.9	3.9	19.0	6.2	18.9	4.1	19.5	5.0	81.4	15.2
Living area										
Large city^a^	23.1	4.8	17.9	6.4	18.8	5.0	18.8	6.2	78.6	18.1
Large town	22.9	4.8	17.3	6.7	18.6	5.2	18.2	6.4	77.0	18.9
Town	22.6	5.1	17.0	6.7	18.9	4.8	18.1	6.5	76.5	18.9
Village/countryside	22.7	5.0	17.4	6.7	19.5	4.7	18.5	6.3	78.1	18.1
Abroad	24.1	4.1	17.6	5.7	18.9	5.1	20.7	6.0	81.3	16.4

^a^Stockholm, Gothenburg and Malmoe

Younger and older age groups tend to generally score higher, and there was a significant mean difference between the age groups [F (5, 2447) = 22.17, *p* = 0.001] on the overall summary FACT-GP mean score. A mean difference of approximately 5 on each subscale indicates a significant difference at *p* < 0.01.

Furthermore, there was a significant difference between income groups on the overall summary FACT-GP mean score [F (6, 2499) = 40.31, *p* = 0.001] and a Bonferroni corrected post hoc comparison showed that higher income was associated with a higher FACT-GP score for each income group (*p* < 0.01). Similarly, a higher level of educational attainment was related to a significantly higher overall summary FACT-GP mean score [F (5, 2595) = 3.97, p = 0.001]. There where however no significant differences between groups when comparing scores between birthplace and living area, even when controlling for gender and age (*p* > 0.05).

### SRH

The eleven-point scale of SRH showed a mean value of 7.20 and a standard deviation of 2.02. Table 5 show Pearson correlations between each subscale and the overall summary FACT-GP scale together with SRH**.** The results are robust when controlling for age, gender, education and income [R^2^ = .55, F (5, 2404) = 582.24, *p* < 0.001].

**Table 5:** Pearson correlation between each subscale, the total FACT-GP scale and the rating-scale of self-perceived health (SRH)

	(1)	(2)	(3)	(4)	(5)	(6)
1. PWB						
2. SWB	.34^***^					
3. EWB	.64^***^	.37^***^				
4. FWB	.69^***^	.58^***^	.64^***^			
5. FACT-GP	.79^***^	.75^***^	.79^***^	.91^***^		
6. SRH	.68^***^	.43^***^	.59^***^	.71^***^	.73^***^	

* *p* < .05, ** *p* < .01, *** *p* < .001

### Cancer diagnosis

Of the respondents, 7.5% (*n* = 198) reported that they had previously been diagnosed with cancer and that they received the diagnosis 0–5 years ago (49.5%), 6–10 years ago (25.8%) or over 10 years ago (24.7%). There was no significant difference between participants who had been diagnosed with cancer (M = 78.78, SD = 18.81) and not (M = 77.63, SD 18.45) on the FACT-GP score (*p* = 0.40). There was furthermore a non-significant tendency that those who had been diagnosed 0–5 years ago (M = 77.14, SD = 18.83), scored relatively lower compared to 6–10 years ago (M = 79.42, SD = 21.49) and more than 10 years ago (M = 81.39, SD = 17.76) (*p* = .43).

Similarly, when analysing SRH there was no significant difference between participants who had been (M = 6.94, SD = 2.21) and those who had not been (M = 7.22, SD 2.00) diagnosed with cancer (*p* = 0.07). There was no significant difference in time since diagnosis on self-rated health (*p* = .41), but the same pattern emerged as in FACT-GP where those who had been diagnosed 0–5 years ago (M = 6.73, SD = 2.29), scored lower compared to 6–10 years ago (M = 7.12, SD = 2.27) and more than 10 years ago (M = 7.18, SD = 1.97).

## Discussion

The aim of this study was to describe HRQOL in the general Swedish population using the FACT-GP instrument and a single item question concerning SRH. To our knowledge this is the largest sample (*n* = 2791) using the FACT-GP instrument and the results could hopefully serve as a normative benchmark against which other HRQOL-data could be given meaning.

Studies have shown that HRQOL varies between countries and cultures, suggesting an important need to provide normative data from different cultures [16, 17]. Few studies have nonetheless described HRQOL in a general population, using either FACT-G or FACT-GP. These existing studies are based on data from US [12], Austria [4] and Australia [7]. The study by Brucker et al. [12] was performed on 1075 participants and compared FACT-G between a general population and a cancer population. The studies by Holzner and colleagues [4] and by Janda and colleagues [7] were based on 968 and 2727 participants respectively. Both last-mentioned studies compared FACT-GP scores with sociodemographic data.

### HRQOL

The present Swedish population scored lower on FACT-GP in comparison with the other studies using FACT-GP to assess HRQOL (M 80.1 [12], M 85.9 [7] and M 86.5 [4]). This may be surprising since the present sample is skewed towards a higher socioeconomic status which would rather indicate a higher HRQOL score. There is no obvious explanation for the observed differences in FACT-GP score between the general populations of those countries studied. When comparing the present results to those of a study investigating different countries’ reference values for the HRQOL-instrument EORTC QLQ-C30 [5], that study show similar results as the present study indicating a lower global health/QoL scores for the Swedish population as compared to the Austrian [5]. Though the results were not in line with the present study when showing higher global health/QoL scores for the US population as compared to the Swedish [5].

### Cancer

The results showed that there was no significant difference in HRQOL as assessed by FACT-GP, between participants who had been diagnosed with cancer and those who had not. The study by Holzner and colleagues [4] showed inconclusive results concerning HRQOL for patients with a previous cancer diagnosis in complete remission in comparison to the general population. That study reported similar FACT-GP scores in comparison to the general population for patients with previous breast cancer, but lower FACT-GP scores for patients with previous bone-marrow transplantation and higher FACT-GP scores for patients with Hodgkin’s disease [4]. In the study by Janda [7] it appears that there was no difference in FACT-GP score between participants with a previous cancer diagnosis (M = 83.9 SD = 15.6) and those without (M = 86.2 SD = 15.0). However the prevalence of cancer was lower in the present study (M = 78.78, SD = 18.81) where in the study by Janda [7], 3% of the sample reported a previous cancer diagnosis as compared to 7.5% in the present study. No details concerning type of cancer or treatments given are available in either of the studies, making deeper comparisons impossible. There is a trend towards increasing HRQOL in cancer patients over time. This pattern has been shown previously for example in malignant melanoma, where the greatest decline in HRQOL is observed at the time of diagnosis and the immediate post-treatment period, but after that, HRQOL slowly improves over time [18, 19].

### SRH

To our knowledge, no previous study has investigated the correlation between SRH and FACT-GP, and the present results show a strong correlation that is robust even when controlling for age, gender, education and income. Though not studying SRH specifically, Holzner et al. [9] showed that participants suffering from chronic illnesses reported lower HRQOL on all subscales and overall FACT-GP as compared to healthy subjects. The study by Janda et al. [7] investigated the effect of co-morbidities and found that HRQOL decreased with increasing number of co-morbidities for all subscales and also the overall FACT-GP score. The strong positive association between SRH and HRQOL is primarily related to better health outcomes [20, 21] and it is believed that health outcomes are associated with healthy behaviours such as regular physical activity, less sedentary time, a healthier diet and non-smoking habits [22]. When interpreting SRH elicited from a general population particularly in relation to a disease-specific population, many studies have noted that patients tend to rate their health to be higher than members of the general population, thus indicating that patients rate their own health higher than members of the general public [23, 24]. In effect, the importance of considering the values reported both by patients and by those of the general populations when interpreting HRQOL- and SHR has been argued by several researchers [23, 25].

### Gender

The present results showed a statistically significant difference between men and women in the overall FACT-GP score, although this difference should be interpreted with caution since the effect size was small. A lower overall reported HRQOL by females was also seen in the Austrian study [4], where women reported significantly lower QOL values than men in the subscales PWB and EWB. A suggested explanation for the differences in that study was that men were potentially less inclined to admit an impaired QOL as compared to women [4]. A similar gender difference has been observed when using also other HRQOL-instruments [26, 27]; it should however be mentioned that a previous study investigating HRQOL using FACT-GP observed no gender differences [7].

### Age

The present results show that younger (< 25 years) and older age groups (> 45 years) tend to score higher in general. The observed results are not coherent with previous FACT-GP results, where the Austrian sample showed a trend of declining HRQOL with increasing age, and where the oldest age group (> 70 years) had significantly lower HRQOL than the younger ages [4]. The study investigating FACT-G scores in the Australian sample did not find any significant differences between different age groups concerning HRQOL [7]. However, the present results are in line with a recently published study investigating country reference values for HRQOL using the HRQOL instrument EORTC QLQ-C30 [5]. The study showed higher global health/QoL scores for younger ages (18–29 years) as well as for higher ages (≥60 years for females and ≥ 70 years for males) as compared to middle ages (30–59 years) for both genders [5].

### Income

The present results showed a trend towards an increasing HRQOL with increasing income, and to our knowledge no other study has investigated this correlation using FACT-GP. Previous studies have investigated if there is a correlation between occupational status and HRQOL and in the Austrian study [4] the participants’ occupational status did not have an effect on any of the FACT-GP subscales. In contrast, the participants working full-time reported higher HRQOL on FWB and overall summary FACT-GP score as compared to participants working part-time in the Australian study [7]. Other studies have shown that wealth and SRH have a strong positive correlation [28] and that higher income is more commonly associated with a positive evaluation of life, including well-being and life satisfaction [6].

### Education

The present results indicated that participants who reported a higher level of completed education had a significantly higher overall summary FACT-GP score, and the same results were observed in both the Austrian [4] and the Australian study [7]. The observed results are probably explained by the fact that more highly educated people are likely to have more satisfying work situations and generally to have more privileged lifestyles [4].

### Birthplace

The present results did not show any significant differences between groups when analysing HRQOL-scores depending on birthplace. Thus, the present study indicates that overall FACT-GP score is not affected by ethnicity. It is not known if our results are in line with other studies since, to our knowledge, no other study has investigated the correlation with ethnicity by investigating location of birth in relation to current country of residency. In general, studies using instruments other than FACT-GP show inconclusive results concerning ethnicity and HRQOL [29, 30].

### Living area

The present results did not show any significant differences between groups when analysing HRQOL-scores depending on living area, such as countryside, small or large towns or abroad. The results concerning living area are in line with the previous study with Australian participants showing no effect on HRQOL [7].

### Strengths and limitations

The main limitation of this study is that the participants had a somewhat higher age, educational level and income compared to the Swedish general population [15], thus indicating a risk for selection bias. As can be seen in Table 1, the sample is representative compared of the Swedish population regarding age and gender, although the youngest and oldest age groups are slightly underrepresented in our sample. The sample is less representative concerning educational attainment especially in the range of lower level education. Mean income in Sweden 2017 was approximately 294′ SEK and the median income was 270′ SEK [31]. The most common reported income level in our data was a monthly salary of between 30,000 and 36,999 SEK. The sample thus showed a higher level of socioeconomic status compared to the Swedish population in general. People with lower living standards are generally hard to reach with surveys even when a randomized postal survey with incentives is employed [32]. To our knowledge, the web panel at the University of Gothenburg is still one of the most representative web panels in Sweden and in some respects may provide a more representative sample compared to traditional postal or telephone surveys. Other limitations are that the present study is a cross-sectional study and that any conclusions concerning causality cannot be drawn, and that there are most probably several confounding factors that are not accounted for in the analysis concerning predictive validity with regard to sociodemographic factors and SRH.

## Conclusions

HRQOL is frequently used as an important endpoint in healthcare research [33, 34] and there is an increasing need for normative data. Since HRQOL could be in part culturally dependent, the present result could hopefully serve in Western cultures as a general population standard against which other HRQOL-data could be given meaning.

## Supplementary information

**Additional file 1: Supplementary Table A1.** Normative data of a Swedish general population sample based on pro-rated mean scores of the 21 items

## Data Availability

Not applicable.

## Abbreviations

EWB: Emotional well-being
FACT: Functional Assessment of Cancer Therapy ()
FACT-G: Functional Assessment of Cancer Therapy - General
FACT-GP: Functional Assessment of Cancer Therapy - General Population
FWB: Functional well-being
HRQOL: health-related quality of life
PRO: patient reported outcomes
PWB: Physical well-being
SRH: self-rated health
SWB: Social/family well-being
