# Supplementary material for: Health-related quality of life (FACT-GP) in Sweden
Source: Health Qual Life Outcomes. 2020 Jun 8;18:172. doi: 10.1186/s12955-020-01420-1 (PMC7278202; doi:10.1186/s12955-020-01420-1)
Supplement: Supplementary file 1 — Additional file 1: Supplementary Table A1. Normative data of a Swedish general population sample based on pro-rated mean scores of the 21 items [file 12955_2020_1420_MOESM1_ESM.docx]

**Supplementary Table A1.** Normative data of a Swedish general population sample based on pro-rated mean scores of the 21 items.

| Item code | Total | | | Males | | | Females | | |
| --- | --- | --- | --- | --- | --- | --- | --- | --- | --- |
|  | M | SD | n | M | SD | n | M | SD | n |
| GP1 | 2.5 | 1.2 | 2683 | 2.6 | 1.1 | 1357 | 2.3 | 1.2 | 1261 |
| GP2 | 3.6 | 0.8 | 2680 | 3.7 | 0.6 | 1353 | 3.5 | 0.9 | 1262 |
| GP3 | 3.5 | 0.9 | 2682 | 3.6 | 0.9 | 1356 | 3.4 | 1.0 | 1263 |
| GP4 | 3.1 | 1.1 | 2683 | 3.2 | 1.0 | 1357 | 2.9 | 1.2 | 1262 |
| GP6 | 3.3 | 1.0 | 2683 | 3.4 | 0.9 | 1354 | 3.2 | 1.0 | 1264 |
| GP7 | 3.7 | 0.7 | 2677 | 3.8 | 0.6 | 1353 | 3.6 | 0.8 | 1259 |
| GS1 | 2.4 | 1.2 | 2676 | 2.3 | 1.2 | 1359 | 2.5 | 1.2 | 1262 |
| GS2 | 2.8 | 1.2 | 2669 | 2.7 | 1.2 | 1355 | 2.8 | 1.1 | 1259 |
| GS3 | 2.4 | 1.2 | 2671 | 2.3 | 1.2 | 1356 | 2.6 | 1.2 | 1260 |
| GS6 | 3.1 | 1.2 | 2651 | 3.0 | 1.2 | 1351 | 3.1 | 1.1 | 1246 |
| GS7 | 1.7 | 1.4 | 2379 | 1.6 | 1.4 | 1277 | 1.9 | 1.5 | 1064 |
| GE1 | 2.9 | 1.2 | 2671 | 3.1 | 1.1 | 1360 | 2.7 | 1.2 | 1264 |
| GE4 | 3.1 | 1.1 | 2669 | 3.2 | 1.1 | 1359 | 2.9 | 1.2 | 1263 |
| GE5 | 3.6 | 0.9 | 2665 | 3.6 | 0.7 | 1357 | 3.5 | 0.9 | 1261 |
| GE6 | 3.1 | 1.1 | 2666 | 3.2 | 1.0 | 1355 | 3.0 | 1.2 | 1264 |
| GF1 | 3.2 | 1.0 | 2654 | 3.3 | 0.9 | 1354 | 3.2 | 1.0 | 1258 |
| GF2 | 2.6 | 1.1 | 2654 | 2.7 | 1.1 | 1354 | 2.6 | 1.1 | 1258 |
| GF3 | 2.6 | 1.1 | 2664 | 2.6 | 1.1 | 1359 | 2.7 | 1.1 | 1263 |
| GF5 | 2.4 | 1.3 | 2659 | 2.5 | 1.2 | 1356 | 2.3 | 1.2 | 1261 |
| GF6 | 2.5 | 1.2 | 2659 | 2.6 | 1.1 | 1355 | 2.5 | 1.2 | 1262 |
| GF7 | 2.4 | 1.2 | 2663 | 2.4 | 1.2 | 1358 | 2.4 | 1.3 | 1263 |
